# Management of Radial Nerve Lesions after Trauma or Iatrogenic Nerve Injury: Autologous Grafts and Neurolysis

**DOI:** 10.3390/jcm9123823

**Published:** 2020-11-26

**Authors:** Karl Schwaiger, Selim Abed, Elisabeth Russe, Fabian Koeninger, Julia Wimbauer, Hassan Kholosy, Wolfgang Hitzl, Gottfried Wechselberger

**Affiliations:** 1 Department of Plastic, Reconstructive and Aesthetic Surgery, Hospital of St. John of God (Barmherzige Brüder) Salzburg, Paracelsus Medical University, Kajetanerplatz 1, 5020 Salzburg, Austria; selim_abed@hotmail.com (S.A.); elisabeth.russe@bbsalz.at (E.R.); fabian.koeninger@bbsalz.at (F.K.); julia.wimbauer@bbsalz.at (J.W.); drhkholosy@gmail.com (H.K.); gottfried.wechselberger@bbsalz.at (G.W.); 2 Department of Plastic Surgery and Reconstructive Surgery, Faculty of Medicine, Alexandria University, Alexandria 21563, Egypt; 3 Research Office-Biostatistics, Paracelsus Medical University, 5020 Salzburg, Austria; wolfgang.hitzl@pmu.ac.at

**Keywords:** radial nerve lesion, nerve regeneration, nerve reconstruction, sural nerve graft, neurolysis

## Abstract

Background: Proximal radial nerve lesions located between the brachial plexus and its division into the superficial and deep branches are rare but severe injuries. The majority of these lesions occur in association with humerus fractures, directly during trauma or later during osteosynthesis for fracture treatment. Diagnostics and surgical interventions are often delayed. The best type of surgical treatment and the outcome to be expected often is uncertain. Methods: Twelve patients with proximal radial nerve lesions due to trauma or prior surgery were included in this study and underwent neurolysis (*n* = 6) and sural nerve graft interposition (*n* = 6). Retrospective analysis of the collected patient data was performed and the postoperative course was systematically evaluated. The Disabilities of the Arm, Shoulder, and Hand (DASH) and the LSUHS (Louisiana State University Health Sciences) scores were used to determine regeneration after surgery. Comparison between the patients’ and calculated normative DASH scores was performed. Results: All patients had a traumatically or iatrogenically induced proximal radial nerve lesion and underwent secondary treatments. The average time from radial nerve lesion occurrence to surgical intervention was approximately four months (1.5–10 months). Eight patients (66.67%) had a humeral fracture. During follow up, no statistically significant difference between the calculated normative and the patients’ DASH scores was observed. The LSUHS scores were at least satisfactory. Conclusions: Neurolysis or sural nerve graft interposition performed within a specific period of time are the primary treatment options for radial nerve lesions. They should be performed depending on the lesion type. Regeneration to a satisfactory degree was observed in all patients, and the majority achieved full recovery of sensory and motor functions. This was the first study to highlight the efficiency of neurolysis and sural nerve graft interposition as secondary treatment interventions, especially for radial nerve lesions.

## 1. Introduction

Radial nerve lesions at the height of the humerus, between the brachial plexus and the nerves’ division, into the superficial and deep branches are rare but very serious injuries. In most cases, these injuries occur in association with traumatic fractures or are induced iatrogenically during surgery. The most common cause of radial nerve damage due to humeral fractures are contusions to the nerve, which tend to heal in approximately three to 12 months. The spontaneous remission of a primary radialis lesion after a humeral fracture tends to happen in 70 to 87% of cases [1]. Nevertheless, there are cases where no spontaneous remission occurs, and the number of iatrogenically induced radial nerve lesions during osteosynthetic surgery is underestimated.

Clinicians must have a thorough knowledge of the radial nerve’s course and branching in order to provide accurate and timely diagnosis [2]. Patients normally present with loss of wrist and finger extension and sensation at the dorsoradial side of the forearm. Surgical exploration is indicated when there is a clear nerve-specific loss of motor and sensory function and a correlation in imaging studies where a nerve lesion can be located. At the time of first manifestation, anatomic reconstruction should be the primary treatment of choice when the nerve fibers can reach the neuromuscular junction within approximately 18 months. Additionally, selective nerve transfers have been discussed in the literature and are suggested to improve the outcome.

Our aim was to evaluate the outcome of neurolysis and sural nerve graft interposition, in patients at our clinic with proximal radial nerve lesions with significant loss of motor (wrist drop) and sensory functions, as treatment procedures secondary to trauma or prior surgery within the last few years.

## 2. Material and Methods

### 2.1. Study Design

Data extraction was performed using the internal coding system of the hospital. Thirty-three patients had undergone radial nerve surgery within the prior 11 years. The inclusion criteria were: (1) Radial nerve reconstruction using sural nerve graft or neurolysis of the radial nerve; (2) nerve lesion located at the height of the humerus between the plexus and division of the radial nerve into the superficial and deep branches.

Finally, 12 patients met the inclusion criteria. Retrospective analysis of surgical outcomes was performed by follow-up examinations and intensive evaluation and data extraction of the hospitals’ medical reporting system. Six patients were examined at the time of study creation, as the other patients died, were acutely ill, or had moved abroad during the study period. All patients were followed up for at least 3 years postoperatively.

Patients were evaluated using the Disabilities of the Arm, Shoulder, and Hand (DASH) score [3] and the LSUHS (Louisiana State University Health Sciences) grading system. The DASH score subjectively measures the patients’ impairments in terms of symptoms, disabilities, and handicaps. It evaluates symptoms and functional status, including pain, weakness, tingling/numbness, and stiffness and physical, social, and psychological wellbeing [2]. As the DASH score decreases, function improves (100–0). We also used a normative DASH score to compare our results to that in an imagined normal population. All patients had unpredictable trauma; consequently, prior assessment of their radial nerve function was impossible. Therefore, we used the following equations to calculate the normative values as reported by Klum et al. [4].

Male value = (0.16 × age) −3.05; Female value = (0.29 × age) − 4.46.

Furthermore, the LSUHS grading system was used (Table 1). This is a clinical assessment of functional impairment, especially in the radial nerve, wherein the functions of individual muscles are evaluated with a score between 0 and 5 (indicating absent and excellent radial nerve function, respectively). The tested muscles differed depending on whether the patient had a proximal or distal radial nerve lesion. Lesion are defined to be located distally in the Louisiana State University Health Sciences grading system at the height of the branching into the posterior interosseous nerve and superficial radial nerve and further distally. All patients had a proximal nerve lesion and; therefore, only the muscles in the proximal radial nerve lesion scale were evaluated. Similar to other studies, we considered an LSUHS score ≥3 as representing a favorable functional outcome.

The study was approved by the ethical committee of the University of Alexandria, Faculty of Medicine and registered under the number 0304601. Informed consent was obtained from all participants. Ethical guidelines according to the Declaration of Helsinki were respected.

### 2.2. Hypothesis

It is hypothesized that neurolysis and sural nerve graft interposition for reconstruction of the radial nerve provide excellent outcome concerning nerve function without significant limitation in daily activities, compared to healthy individuals in the same age.

### 2.3. Diagnostics

Prior to surgery, an accurate diagnostic workup is essential. Apart from performing clinical examination and documentation of neurologic deficits, pre-and postoperative examinations, including neurography performed by a neurologist, are done. Additionally, sonography or magnetic resonance imaging, which is preferred, is conducted to visualize the nerve. Additionally, radiography is performed when the osteosynthetic material is in situ. The radiologists at our institute usually draw preoperative skin markings at the suspected radial nerve lesion site to facilitate surgical exploration.

For nerve lesions, which were treated with simple neurolysis, nobody knows how function would have improved without surgery. Thus, in our department, we are trying to base our decision regarding intervention or not on an objective basis: Visible nerve damage or irritation on ultrasound or MRI and a bad neurography. If the patient has a full loss of especially motor function combined with a visible nerve damage or irritation on ultrasound or MRI, at least surgical exploration and neurolysis is warranted as early as possible.

Anatomic nerve reconstruction using sural nerve grafts is only indicated when there is a chance for the regenerating axons to reach the neuromuscular junction within about 18 months from the first clinical appearance. Indeed, we rely on the general knowledge regarding the neuroanatomic repair mechanisms. This means that the axons grow 1 mm per day from the site of the lesion to the neuromuscular junction of the muscle due to the Wallerian degeneration of the nerve distal to the lesion. Considering that the most proximal radial nerve lesions are located approximately 10 cm proximal to the first extensor muscles, the first clinical and neurographical improvements would be observed approximately 3.5 to 4 months postoperatively, and these procedures are performed within 10 months to 1 year after nerve injury. In cases where there is no sign of reinnervation at the end of this period, revision surgery, selective nerve transfer, and/or musculotendinous transfer is considered.

### 2.4. Surgical Technique

**Figure 1:**
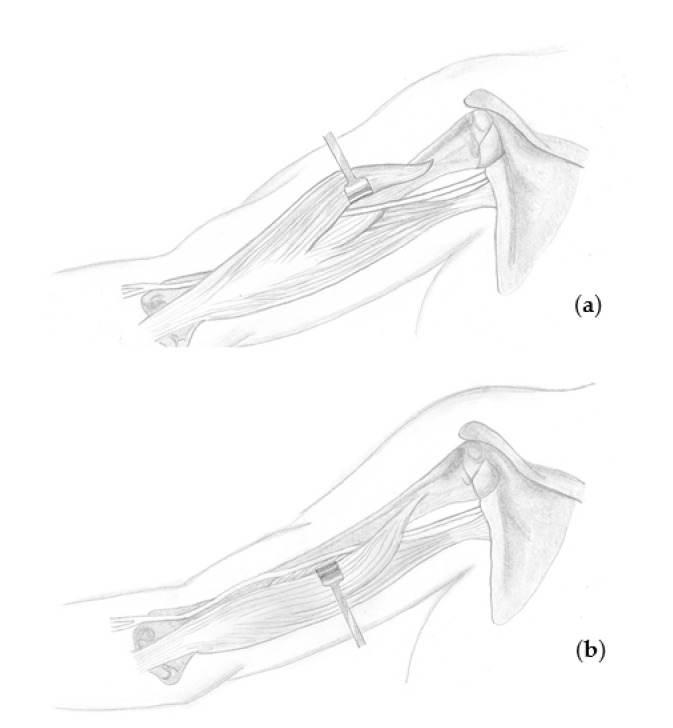
When a radial nerve lesion is suspected to be in the upper third of the humerus, the lateral head of the triceps muscle is identified and retracted medially and anteriorly (**a**) towards the patient to visualize the radial nerve between the medial and lateral heads of the triceps. When the lesion is suspected to be in the middle or lower third of the humerus (until about 10 cm proximally to the elbow), the lateral head of the triceps muscle should be retracted laterally and dorsally (**b**) to identify the nerve between the lateral head and the flexor muscles.

**Figure 2:**
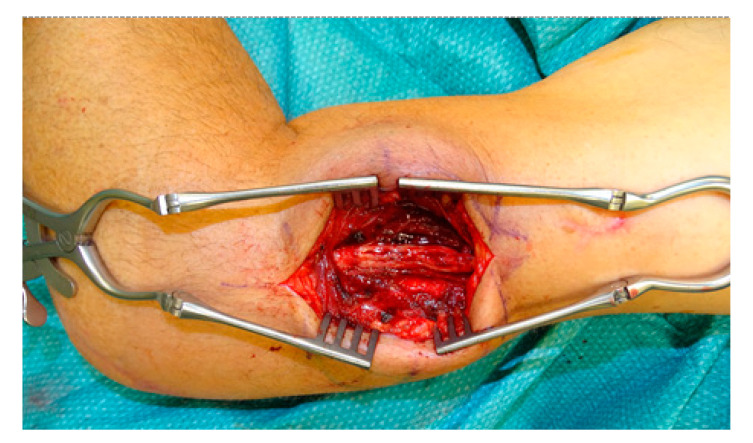
Intraoperative clinical correlation of Figure 1b, showing how to reach the radial nerve best. In this case the lesion was located in the lower third. Reconstruction was done using sural nerve graft interposition.

The patient is placed side-lying with the arms abducted and visible forearm, wrist, and fingers. Surgery is performed under general anesthesia; at the time of nerve stimulation, paralytic agents must be antagonized. When a sural graft is needed, the lateral calf is prepared for the graft harvest. Skin incision is performed at the suspected nerve lesion site, normally at the projection of the posterior-lateral upper arm to the humeral groove based on the old scar in cases where a previous surgery has been performed. Subcutaneous preparation is done using monopolar cautery. When the radial nerve lesion is suspected to be located in the upper third of the humerus, the lateral head of the triceps muscle is identified and retracted medially and anteriorly towards the patient to visualize the radial nerve between the medial and the lateral head of the triceps. When the lesion is suspected to be located in the middle or lower third of the humerus (until about 10 cm proximally to the elbow), the lateral head of the triceps muscle is retracted laterally and dorsally to identify the nerve between the lateral head and the flexor muscles (Figure 1). In the majority of our cases, the damaged nerve is reached through a dorsolateral skin incision, retracting the lateral head of the triceps and exploring the nerve between the flexor and extensor muscle compartment (Figure 2). The site of the suspected lesion is clearly visualized. When the nerve is in continuity without macroscopic and microscopic damage and can be intraoperatively stimulated with the nerve stimulator, only decompression and neurolysis is performed. In contrast, when the nerve is in continuity and cannot be stimulated, the surgeon decides whether only neurolysis or excision of the suspicious nerve area with subsequent sural nerve graft reconstruction is performed. Normally, in cases of no intraoperative stimulation, excision and nerve reconstruction is done. In cases of neuroma or lesion formation with no continuity of the nerve, excision along the nerve ends is done until healthy fascicular structures can be observed and reconstruction of the defect with sural nerve graft reconstruction is performed. Graft interposition is performed from fascicle to fascicle with end-to-end suturing using monofil 9.0 interrupted sutures under microscopic guidance. At the end of the nerve graft interposition, fibrin glue is used for additional stabilization.

**Figure 3:**
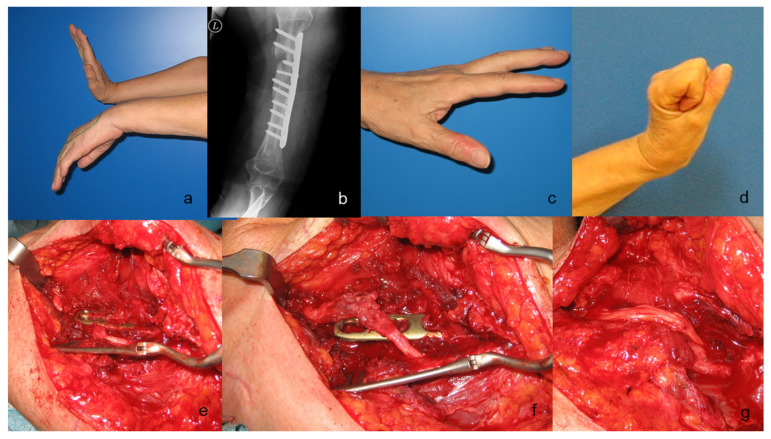
A case of a 67-year-old woman with complete loss of radial nerve function after osteosynthesis of the humerus after a fracture. Preoperative clinical presentation (**a**), preoperative radiograph (**b**), and postoperative result displaying complete radial nerve function recovery at 8 months postoperatively (**c**,**d**). Intraoperative view showing a completely pinched nerve under the osteosynthesis plate (**e**). A screw was removed, and the plate was lifted up to release the nerve, which was completely disrupted (**f**). A decision was made to reconstruct a 5 cm segment of the nerve with four sural nerve grafts (**g**).

**Figure 4:**
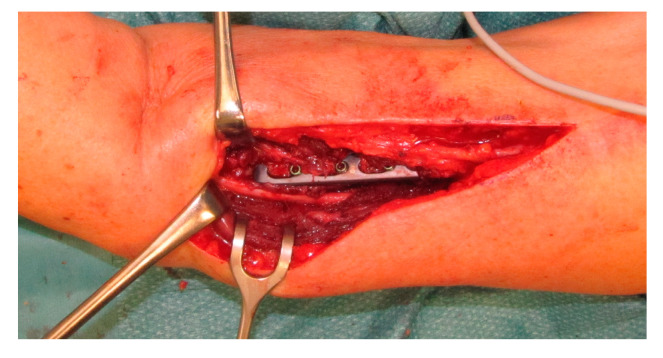
Intraoperative view during revision surgery in a 73-year-old woman who underwent plate implantation due to fracture. She had complete loss of radial nerve function after fracture treatment. Intraoperative exploration showed that the nerve was retracted dorsally by the plate, but it was in continuity.

**Figure 5:**
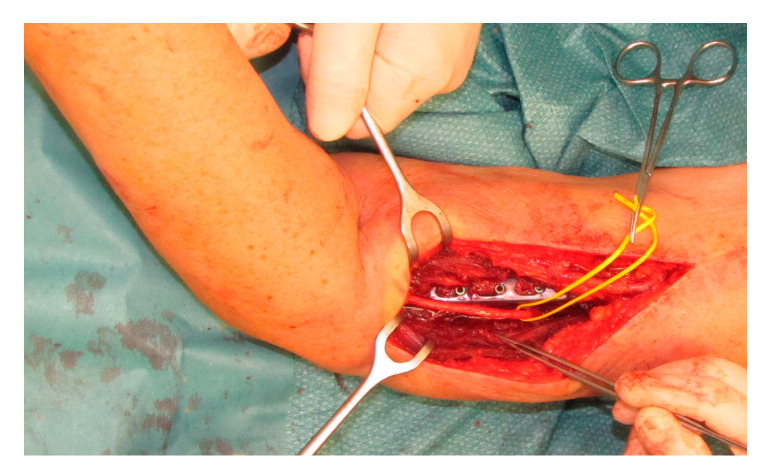
Neurolysis was performed and the patient regained full radial nerve function at 6 months postoperatively.

Figure 3, Figure 4 and Figure 5 present the cases of two patients who were treated with sural graft nerve reconstruction or simple neurolysis.

### 2.5. Statistical Analysis

All statistical analyses were performed using National Council for the Social Service (NCSS) version 10 (NCSS, LLC., Kaysville, UT, USA) and Statistica version 13 (Dell Technologies Inc., Round Rock, TX, USA). The data were checked for consistency, and a dependent bootstrap *t*-test based on 7000 Monte Carlo simulations was used to compare the DASH-scores. The test was two sided, and the statistical significance was set at *p* < 0.05.

## 3. Results

**Figure 6:**
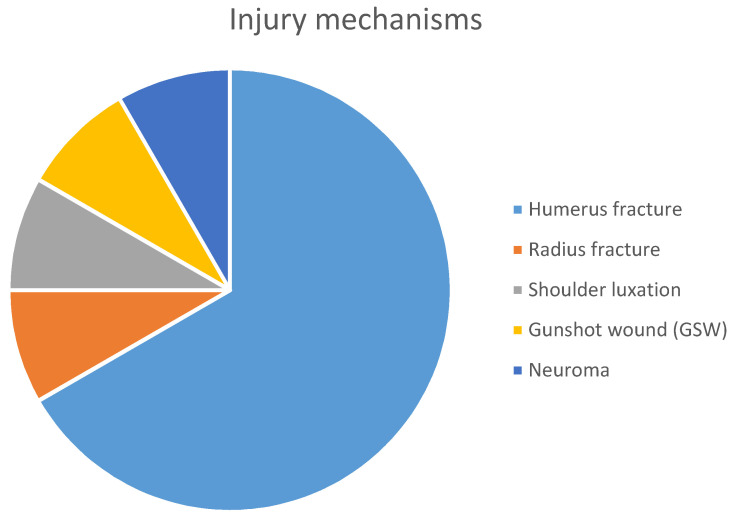
Distribution of injury mechanisms and reasons for radial nerve damage.

There were seven male and five female participants (total, 12; mean age at the time of the surgery, 51.16 years; age range, 7–70 years, standard deviation, 19.97). Nine patients (75%) had fractures (eight patients with a fractured humerus and one with a fractured radius and olecranon). The other three patients had shoulder luxation, a gunshot wound in the upper arm, and neuroma formation due to a previous surgery on the radialis nerve in another hospital. The injury mechanisms are presented in Figure 6.

The average time from radial nerve lesion occurrence to surgical intervention was approximately four months (1.5–10 months). The patients underwent sural nerve graft reconstruction (50%) and neurolysis (50%) (*n* = 6 in each intervention). The average amount of sural nerve grafts used per surgery was three (two to five). The average operative time for patients who underwent sural nerve grafting and neurolysis were 4 h and 20 min and 1 h and 46 min, respectively. Table 2 shows the patient characteristics and collected data.

**Figure 7:**
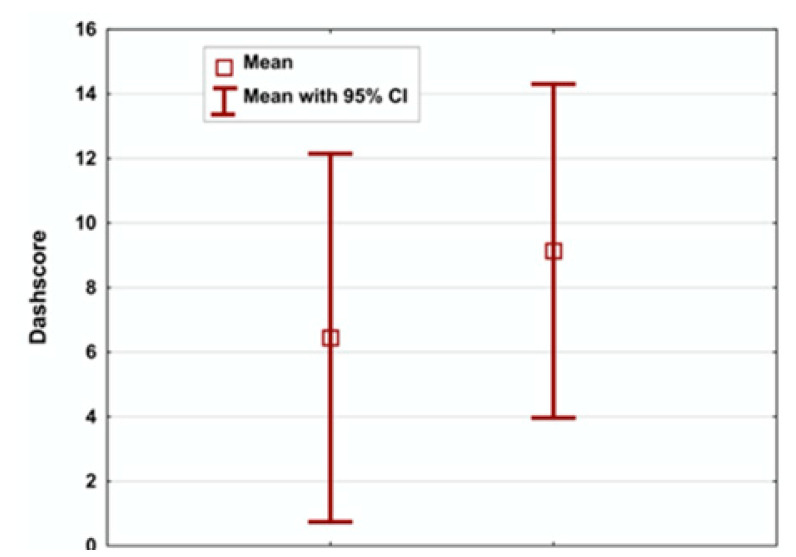
Box plots: the right and the left boxes represent the normative calculated and the examined patients’ Disabilities of the Arm, Shoulder, and Hand (DASH) scores.

Five of the six patients who were followed up had a DASH score <15. One patient had a score of 50. The expected DASH score for healthy individuals of the same age was estimated between 2.23 and 17.87. The examined and the estimated DASH scores are listed in Table 3 and Figure 7.

No statistically significant differences between the measured and estimated DASH scores were observed (*p* > 0.05).

All patients had a LSUHS score ≥3. Especially, two, one, and three patients received a score of 3, 4, and 5, respectively. The subjective patient satisfaction was rated at 100% by all patients. The earliest surgery for the examined patients was performed 10 years prior, indicating that these patients had a regeneration time of three to 10 years before examination.

## 4. Discussion

Neurolysis or nerve reconstruction using nerve grafts are the most commonly recommended treatment options for patients who undergo secondary treatment for radial nerve lesions.

The scientific workup of these lesions is difficult, because no preoperative data of previous function before trauma were available for comparative analysis.

In our work, we used a normative DASH score, which was calculated using an equation reported by Klum et al. [4]. This makes the postoperative results comparable to those of healthy individuals of the same age. The average calculated normative DASH score was approximately 6.5, while the observed average DASH score was 9, with no significant difference. This finding indicates that the postoperative function of our patients was comparable to that of healthy individuals. Moreover, a patient with a DASH score of 50 was considered to be an outlier, as his axillary injury led to an increased DASH score along with his radial nerve reconstruction. We were not able to evaluate his axillary nerve injury using the DASH questionnaire, which led us to exclude his DASH score from our statistical analysis. This patient was; however, included in the LSHUS score analysis.

The LSHUS score has been designed specifically for evaluating radial nerve function. A LSUHS score ≥3 is considered satisfactory, indicating a successful surgery (similar to the findings of numerous other studies on the radial nerve). This score was obtained in all examined patients highlighting the successful completion of surgery.

Our comparative literature analysis focused on nerve reconstruction surgery, especially for the radial nerve and revealed the following: Kim et al. [5] reported encouraging results in patients who required only neurolysis due to positive nerve action potentials (NAP) when lesions in continuity were caused by trauma. Grade 3 or better functional recovery was observed in 21 (95%) of 22 patients in their study. When no NAP was recorded, neuroma resection and suture or graft repair resulted in a favorable outcome in 91% of the patients.

Terzis et al. [6] mentioned that functional recovery was significantly better in patients who underwent neurolysis of the radial nerve. They attributed these results to the fact that neurolysis is generally performed on lesions in continuity and is less invasive than nerve graft interposition.

In general, according to the authors’ opinion, the comparison between neurolysis and sural nerve grafting regarding their outcomes is not useful, as their indications are different. The type of surgery that should be performed is strongly associated with the type of injury and lesion extension. Neurolysis is indicated in cases where the nerve is in continuity without neuroma formation, and it has been shown to improve function and radiological signs of compression. In contrast, sural nerve grafting is indicated when the nerve has lost its continuity and/or a neuroma formation is present, causing loss of function (negative NAP). Normally, in cases of no intraoperative stimulation, we recommend excision and nerve reconstruction. The treatment options for neuromas in continuity also include neurolysis or sural nerve grafting. In these cases, the type of surgery is dependent on the severity of function loss, the neuroma size, and the presence of positive or negative NAPs. Neurolysis, transposition, and/or nerve wrapping should be favored in cases wherein the neuroma is only a local problem inducing pain with or without small neurological deficits.

Murovic [7] found that secondary graft repair for lesions not in continuity had the best outcomes in 68% and 67% of median and radial nerve repair cases, respectively. Singh et al. [8] found that short nerve grafts generally showed better results than long ones In our study, we achieved a 100% success rate in secondary graft repair for radial nerve lesions. Murovic [7] also noted that neurolysis and suture repair achieved good results in 98% and 88% of radial nerve in-continuity lesion cases with positive and negative NAPs, respectively. Moreover, they indicated that 86% of radial nerve in-continuity lesions with negative NAPs treated with graft repair were grade ≥3. Similarly, Terzis et al. [5] reported that good and excellent motor results (M3 to M5) were observed in 77% of patients; this rate is lower than that in our study.

Esquenazi et al. [9] published a series of radial nerve lesion cases treated with neurolysis, direct suture, or sural nerve grafting. Nine patients required only external neurolysis because the lesions were in continuity and a positive NAP recording was obtained across the lesion. All patients received a score ≥ 4 indicating good functional recovery, with some residual weakness in terms of extension of the thumb and fingers. In their study, eight patients with lesions in continuity, but without recorded NAPs, underwent end-to-end suture or graft repair (seven and one patients, respectively) after resection of a 3 cm non-recordable segment. All these patients achieved a functional recovery grade of 3 or 4. These results indicate that it is possible to achieve functional recovery of at least grade 3 in 100% of patients.

Our study has several limitations, including the retrospective design and the single center experience, as well as the small sample size. Nevertheless this is the first study, which could show an extraordinarily good outcome of secondary treated radial nerve lesions. These findings strongly support the statement, that in cases of doubt, revision surgery on the radialis nerve is strongly recommended. Altogether, few studies [6,7,8,9] have examined the postoperative outcomes of proximal radial nerve lesions after trauma or iatrogenic injury that were treated with secondary intervention using sural nerve grafting or neurolysis. These procedures should be performed within an allowable time window to ensure satisfactory functional recovery. Further, regardless of the type of injury and whether a sural nerval graft or only neurolysis was needed, our interventions led to a nearly 100% regeneration rate and satisfactory postoperative functional outcomes. However, it should be noted that the type of surgery depends on the type of lesion, and the lack of standardized testing generally impedes the comparison of results of nerve reconstruction techniques.

## Figures and Tables

**Table 1:** The Louisiana State University Health Sciences grading system.

Grade	Evaluation	Description
Individual muscle grades–proximal radial nerve (RN) lesion		
0	Absent	Absent radial motor function
1	Poor	Trace of contraction in triceps muscle, absence or trace of contraction in Brachioradialis muscle (BRM) against gravity only
2	Fair	Triceps/BRM contraction against force, but absence or trace of supination and no wrist extension
3	Moderate	Triceps and BRM contraction against force; supination and wrist extension against gravity; absence or trace of finger and thumb extension
4	Good	Triceps and BRM contraction against force; supination and wrist extension against force; trace or better finger and thumb extension
5	Excellent	Good triceps/BRM contraction, supination and wrist extension; finger/thumb extension at least against gravity and some resistance
Individual muscle grades–distal RN lesion involving posterior interosseous nerve (PIN)		
0	Absent	Absent extensor carpi ulnaris, extensor communis/extensor pollicis longus muscle function
1	Poor	Trace of contraction in the extensor carpi ulnaris muscle against gravity only; absent extensor communis and pollicis longus muscle function
2	Fair	Recovery of the extensor carpi ulnaris muscle; absence or trace of the extensor communis or the extensor pollicis longus muscle, or both
3	Moderate	Recovery of the extensor carpi ulnaris muscle, some extensor communis muscles, absence or trace of the extensor pollicis longus muscles
4	Good	Full strength of the extensor carpi ulnaris muscle, recovery of the moderate strength of extensor communis and the extensor pollicis longus muscles
5	Excellent	Full recovery of the extensor carpi ulnaris, extensor communis, and the extensor pollicis longus muscles

**Table 2:** Patient characteristics, type of treatment, surgery time, and follow up examinations.

Patient	Age	Trauma (Yes/No)	Injury Mechanism	Side of Injury (Right/Left)	Surgical Technique	Time between Lesion Occurrence and Intervention	Incision to Suture Time	Follow Up (Years)	Examined for Study/Reason for no Examination
1	30	Yes	Gunshot-wound in the left upper arm	Left	Sural nerve graft	10 months	192 min (3–12 h)	3.5	Examined
2	67	Yes	Humerus fracture	Left	Sural nerve graft	2.5 months	163 min (2–43 h)	10	Examined
3	61	Yes	Humerus fracture	Right	Sural nerve graft	2.5 months	574 min (9–34 h)	2.5	Not examined; patient died
4	7	Yes	Humerus fracture	Right	Sural nerve graft	3.5 months	292 min (4–52 h)	1.5	Not examined due to movement
5	36	Yes	Neuroma formation	Left	Sural nerve graft	7 months	217 min (3–37 h)	5	Examined
6	52	Yes	Radial fracture	Left	Sural nerve graft	1.5 months	135 min (2–15 h)	3	Examined
7	72	Yes	Humerus fracture	Left	Neurolysis	1.5 months	62 min (1–02 h)	2.5	Not examined due to acute illness
8	30	Yes	Humerus fracture	Left	Neurolysis	3 months	121 min (2–01 h)	8	Examined
9	69	Yes	Luxation of the shoulder	Right	Neurolysis of the radial nerve, neurolysis of parts of the axillary nerve	5 months	-	10	Examined
10	54	Yes	Humerus fracture	Right	Neurolysis	3 months	72 min (1–12 h)	1	Not examined; patient died
11	72	Yes	Humerus fracture	Left	Neurolysis	2.5 months	169 min (2–49 h)	1.5	Not examined; patient died
12	64	Yes	Humerus fracture	Right	Neurolysis	9 months	-	9	Not examined due to immobility

**Table 3:** Comparison between the normative and examined DASH scores (the higher the DASH score, the better the function; ranging from 0 to 100) after surgery and nerve function rating according to the LSUHS grading system (ranging from 0 to 5 (absent and excellent radial nerve function, respectively)).

Patient	Type of Surgery	Examined DASH Score	Normative DASH Score	LSUHS Score
1	Sural nerve graft	5	2.23	4
2	Sural nerve graft	83.3	17.87	5
5	Sural nerve graft	13.33	3.51	3
6	Sural nerve graft	12.4	5.59	3
8	Neurolysis	14.12	3.03	5
9	Neurolysis	50	9.59	5

DASH, Disabilities of the Arm, Shoulder, and Hand; LSUHS, Louisiana State University Health Sciences.
